# Regulation of Ubiquitin Enzymes in the TGF-β Pathway

**DOI:** 10.3390/ijms18040877

**Published:** 2017-04-20

**Authors:** Prasanna Vasudevan Iyengar

**Affiliations:** Cancer Science Institute of Singapore, National University of Singapore, Singapore 117599, Singapore; csipvi@nus.edu.sg; Tel.: +65-6516-5995

**Keywords:** transforming growth factor-β (TGF-β), ubiquitin, deubiquitylating enzymes (DUBs), SMAD ubiquitination regulatory factor (SMURF), ubiquitin–proteasome system (UPS)

## Abstract

The transforming growth factor-β (TGF-β) pathway has a tumor suppressor role in normal and premalignant cells but promotes oncogenesis in advanced cancer cells. Components of the pathway are tightly controlled by ubiquitin modifying enzymes and aberrations in these enzymes are frequently observed to dysregulate the pathway causing diseases such as bone disorders, cancer and metastasis. These enzymes and their counterparts are increasingly being tested as druggable targets, and thus a deeper understanding of the enzymes is required. This review summarizes the roles of specific ubiquitin modifying enzymes in the TGF-β pathway and how they are regulated.

## 1. Introduction

Transforming growth factor-β (TGF-β) is a multi-functional cytokine which is involved at key stages of embryogenesis and adult tissue homeostasis [1]. The TGF-β pathway plays crucial roles in regulating important cellular events such as apoptosis, cell proliferation, epithelial-mesenchymal transition (EMT), cytostasis, cellular migration, etc. in a context- or cell-type-dependent manner [2]. More importantly, in normal and premalignant cells, TGF-β pathway elicits a tumor suppressive role. Unfortunately, in advanced metastatic tumors, the pathway is hijacked by cancer cells to promote oncogenic behavior [3,4]. This switch in roles from a “tumor suppressor” to an “oncogene” has been extensively studied in the past few years and has been attributed to differential expression patterns of downstream target components or changes in binding partners of cytoplasmic effector molecules. The direct inhibition of key components of the TGF-β pathway as a therapy has been used to control advanced cancers with concerns that normal or premalignant cells might lose the tumor suppressor arm of TGF-β pathway. One of the ways to overcome this challenge would be to target components that are aberrantly expressed in tumors that have been known to dysregulate the pathway. In recent years, components of the ubiquitin–proteasome system (UPS) have been well studied in the TGF-β pathway and have been shown to have significant roles governing the overall signaling output. These components are being increasingly tested as druggable targets that can pave the way for personalized therapy or potential prognostic biomarkers. Although several ubiquitin enzymes are known to act upon different components of the TGF-β pathway, this review summarizes the enzymes that have been shown to become regulated by other post-translational modifications (PTMs) within the context of the TGF-β pathway. Having this knowledge should give us a deeper understanding of the ubiquitin enzymes, which may be potentially targeted in human diseases.

## 2. Ubiquitin and Ubiquitin-Like Proteins

Ubiquitin is a highly conserved 76 amino-acid polypeptide which is conjugated onto specific proteins or substrates via sequential steps termed “ubiquitylation” [5]. The process is initiated when an E1 ubiquitin-activating enzyme activates a ubiquitin molecule in an ATP (adenosine triphosphate)-dependent manner. Activated ubiquitin is then transferred to an E2 conjugating enzyme which ultimately conjugates it to E3 ubiquitin ligases. The E3 ubiquitin ligases confer substrate specificity and transfer ubiquitin onto lysine residues of substrates, thus monoubiquitylating it. Ubiquitin itself has seven lysine residues K6, K11, K27, K29, K33, K48, and K63 onto which ubiquitin can be attached forming a chain, a process known as polyubiquitylation. K48 linked polyubiquitin chains mark its substrates for degradation via a proteasome, whereas K63 linked polyubiquitin chains serve non-degradative functions such as signaling or protein trafficking [6]. Other lysine-linked polyubiquitin chains are broadly referred to as “atypical” polyubiquitin chains and their functions are beginning to emerge [7]. More than 500 E3 ubiquitin ligases are known to exist in the human genome and some of them are well-characterized to play essential roles in the TGF-β pathway. The E3 ubiquitin ligases are broadly classified according to their functional catalytic domains such as HECT (homologues to E6-associated protein carboxyl terminus), RING (really interesting new gene), and Cullin. Ubiquitylation itself is a reversible process which is mediated by a group of enzymes called deubiquitylating enzymes (DUBs); just over a hundred DUBs are known to exist in the human genome. Apart from ubiquitin, other similar-sized proteins, commonly referred to as “ubiquitin-like proteins,” such as SUMO (small ubiquitin-like modifier) and NEDD8 (neural precursor cell expressed developmentally down-regulated protein 8) have also been well studied. These ubiquitin-like proteins are conjugated onto its substrates in a manner similar to ubiquitylation and their functions in the TGF-β pathway are beginning to emerge.

## 3. The Canonical TGF-β (Transforming Growth Factor-β) Pathway

**Figure 1:**
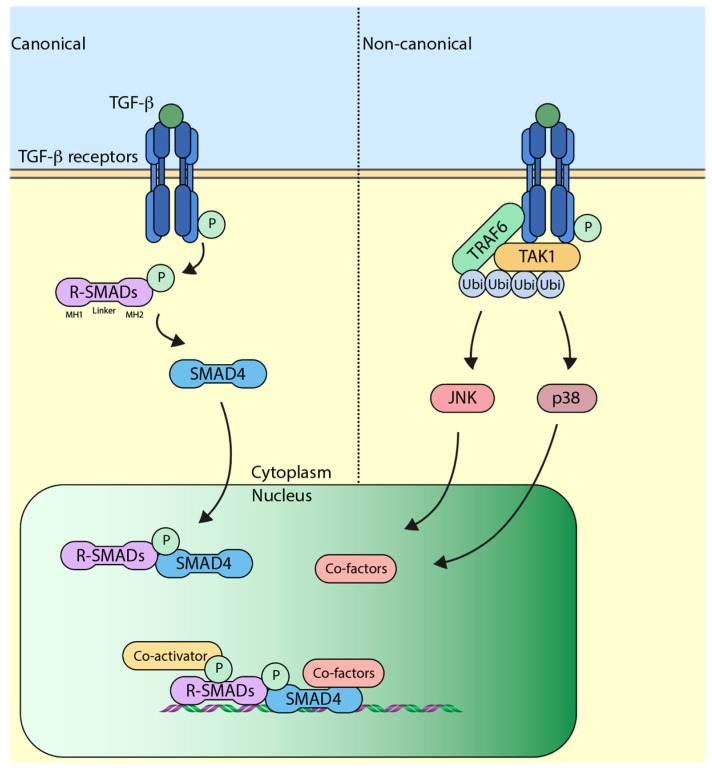
Schematic representation of the transforming growth factor-β (TGF-β) pathway. Left-hand side of the dotted line represents canonical pathway, wherein signaling is transmitted by a SMAD (sma and mothers against decapentaplegic)-dependent mechanism, whereas the right-hand side represents non-canonical pathway, which is SMAD-independent. R-SMADs: Regulatory elements SMADs, Ubi: Ubiquitin, JNK: c-Jun N-terminal kinase, TAK1: TGF-β associated kinase 1, TRAF6: TNF receptor-associated factor 6, P: Phosphate group.

Binding of TGF-β ligand to TGF-β receptors initiates a transphosphorylation event whereby TGF-β receptor II (TβRII) phosphorylates TGF-β receptor I (TβRI) at serine/threonine sites. This event triggers a downstream intracellular signaling cascade mediated by SMAD (sma and mothers against decapentaplegic) regulatory elements (R-SMADs) which include SMAD2 and SMAD3. Once phosphorylated by the receptor, SMAD2/3 form a complex with SMAD4 (Co-SMAD) and translocate to the nucleus [8]. The SMAD proteins have mad-homology 1 and 2 (MH1 and 2) domains at its N and C terminus, respectively, which is separated by a linker region. The MH1 domain has the ability to recognize specific DNA sequences, whereas the MH2 domain is responsible for mediating protein–protein interactions [9]. Once inside the nucleus, the SMAD2/3-SMAD4 complex assemble other transcriptional co-factors and initiate transcription of TGF-β target genes (Figure 1). Interestingly, this also induces the expression of SMAD7, which binds to the receptor and negatively regulates the signaling [10,11]. SMAD7 has been shown to recruit the E3 ubiquitin ligase SMURF2 (SMAD ubiquitination regulatory factor 2) to the receptor, which ubiquitylates and causes its proteasome-dependent degradation, which eventually leads to the attenuation of TGF-β signaling [12]. Around the same time that SMURF2 was characterized to play a role, SMURF1, which is a closely related protein, was also found to attenuate the TGF-β signaling in a similar manner [13]. Apart from SMURF1/2, another E3 ubiquitin ligase that has been shown to influence the canonical TGF-β pathway is NEDD4-L (NEDD4-Like). Inside the nucleus, the linker region of SMAD2/3 is phosphorylated by CDK8 and CDK9 (cyclin-dependent kinase 8/9), which enhances the transcriptional activity of SMAD2/3 by favoring binding to other transcriptional co-activators [14]. Furthermore, the phosphorylated residues also prime SMAD2/3 for another round of phosphorylation by GSK3 (glycogen synthase kinase 3), which is recognized by the WW domain of NEDD4-L [15,16]. Recognition of NEDD4-L causes the ubiquitin-mediated degradation of SMAD2/3, thereby attenuating the signaling [17]. Previous studies have suggested that recognition of SMAD proteins by E3 ubiquitin ligases is driven by WW domains, and this ability is impaired upon phosphorylation of WW domains [18]. It is important to note that, although structurally similar to SMAD2/3, the binding of SMAD7 to WW domains of SMURF1/2 or NEDD4-L is not dependent on phosphorylation [19].

## 4. The Non-Canonical TGF-β Pathway

In contrast to canonical TGF-β pathway, which is SMAD-dependent signaling, SMAD-independent non-canonical TGF-β signaling has also been widely studied. The non-canonical pathway is carried out by other intracellular signaling molecules, such as TAK1 (TGF-β associated kinase 1), p38 MAPK (mitogen-activated protein kinase), and JNK (c-Jun N-terminal kinase) [20,21]. Upon TGF-β ligand binding to the receptors, non-canonical signaling is initiated when TRAF6 (TNF receptor-associated factor 6) binds to the receptors, which causes its autoubiquitylation, which in turn activates TAK1 [22]. Activated TAK1 initiates downstream signaling events comprising of p38 and JNK (Figure 1). The role of SMAD6 as a negative regulator of non-canonical signaling has also been studied. SMAD6 has been shown to recruit A20, a deubiquitylating enzyme that removes ubiquitin off TRAF6 and deactivates it; this ultimately leads to the attenuation of non-canonical TGF-β signaling [23].

## 5. Interplay of Ubiquitylating Enzymes and DUBs (Deubiquitylating Enzymes)

### 5.1. USP15 (Ubiquitin-Specific Protease 15) Targets SMURF2 (SMAD Ubiquitination Regulatory Factor 2) for Deubiquitylation and Deactivates It

**Figure 2:**
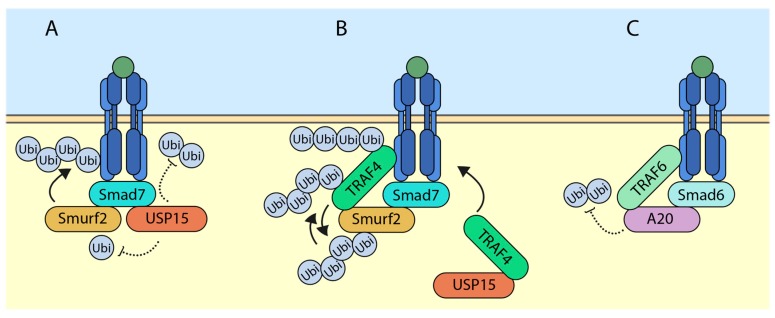
Ubiquitylating enzymes and deubiquitylating enzymes (DUBs) govern the outcome of TGF-β signaling. (**A**) USP15 deubiquitylates both TGF-β receptor and SMURF2 (SMAD ubiquitination regulatory factor 2) to enhance the signaling; (**B**) TRAF4 can either ubiquitylate itself or SMURF2 forming different polyubiquitin chains, and it has been shown to recruit USP15 (Ubiquitin-specific protease 15) to the receptor; (**C**) SMAD6 recruits A20 to the receptor which deubiquitylates TRAF6 and attenuates the signaling. Arrows indicate addition and dotted lines indicate removal.

Ubiquitin-specific protease 15 (USP15) was previously shown to deubiquitylate the TGF-β receptors and rescue it from SMURF2-mediated degradation [24]. The study further showed that USP15 was a positive regulator of the TGF-β signaling. In advanced breast cancer and glioblastoma, higher levels of expression of USP15 correlated with higher overall TGF-β signaling. At the molecular level, USP15 was shown to bind to SMAD7 and exist in a complex with SMURF2. In this scenario, SMURF2 and USP15 had opposite effects on TGF-β receptor stability. Interestingly, we have shown that USP15 also had the ability to regulate the activity of SMURF2 (Figure 2A) [25]. Among other DUBs, which have been previously shown to regulate TGF-β receptor levels, USP15 was shown to deubiquitylate SMURF2. In the study, through mass spectrometric analysis, several ubiquitin acceptor lysine sites were scored. Among these, USP15 was able to remove ubiquitin on SMURF2 at K345, K412, K615, K620, K687, and K734. Interestingly, mutating the lysine 734 of SMURF2 inhibited the ability of SMURF2 to attenuate TGF-β signaling. With the help of molecular docking, it was found out that ubiquitin at lysine 734 of SMURF2 was critical for its activity and USP15 was able to regulate this site, hence affecting its overall function.

### 5.2. TRAF4 (TNF Receptor-Associated Factor 4) Ubiquitylates and Degrades SMURF2

Recently, Long Zhang and colleagues have characterized the function of TRAF4 (TNF receptor-associated factor 4) in regulating TGF-β mediated signaling in EMT [26]. Upon TGF-β stimulation, TRAF4, a member of the RING domain containing E3 ubiquitin ligase, binds to TGF-β receptors, which triggers its lysine-63-linked polyubiquitylation, whereas a TRAF4 mutant lacking a RING domain was unable to ubiquitylate, indicating that TRAF4 causes its autoubiquitylation. These polyubiquitin chains were required to recruit TAK1 which caused its activation. The study further identified SMURF2 as a novel interacting partner of TRAF4 (Figure 2B). Overexpression of TRAF4 decreased the protein levels of SMURF2, which was rescued by the proteasome inhibitor MG132. Furthermore, TRAF4 wildtype but not the catalytically inactive mutant significantly increased the polyubiquitylation of SMURF2. Interestingly, when tagged TGF-β receptors where immunoprecipitated, receptor-associated TRAF4 was also found to be ubiquitylated by SMURF2. This indicates the ability of TRAF4 and SMURF2 to ubiquitylate and antagonize each other. However, previous studies of TRAF family members antagonizing SMURF1/2 and vice versa have been reported [27,28,29]. It was also noted that TRAF4-deficient cells had a higher expression of E-cadherin, a marker for epithelial state of cells, suggesting the involvement of TRAF4 in TGF-β mediated EMT. Additionally, the depletion of TRAF4 enhanced cell–cell adherence, reduced cell migratory capacity, and reduced changes in EMT.

### 5.3. SMAD6 (Sma and Mothers against Decapentaplegic 6) Recruits A20 to Deubiquitylate TRAF6

The TGF-β pathway also has the ability to initiate SMAD-independent signaling, otherwise known as the “non-canonical” pathway. In the non-canonical pathway, upon TGF-β ligand stimulation, receptors recruit TRAF6, which leads to lysine-63-linked autoubiquitylation, which in turn recruits and activates TAK1 [22]. This initiates a chain of intracellular signaling events, which leads to activation of p38 and JNK [30]. Furthermore, an interesting study uncovered a novel mechanism through which SMAD6 acts as a negative regulator for the non-canonical TGF-β induced signaling [23]. In AML12 (α mouse liver 12) cells, upon SMAD6 knockdown and TGF-β stimulation, TRAF6 polyubiquitylation was sustained much longer than control cells. Conversely, the overexpression of SMAD6 inhibited lysine-63-linked polyubiquitylation of TRAF6 in a dose-dependent manner. The authors go on to show that a deubiquitylating enzyme, A20 was responsible for the SMAD6-mediated deubiquitylation of TRAF6 (Figure 2C). At the molecular level, SMAD6 acts as a mediator between A20 and TRAF6 interaction. Deletion mutation analysis revealed that SMAD6 bound TRAF6 and A20 via its MH2 and linker regions, respectively. The activation of TGF-β signaling through TRAF6-TAK1 has been linked to induction of apoptosis; further experiments revealed that knockdown of either SMAD6 or A20 led to increase in TGF-β-mediated apoptosis in AML12 cells due to sustained levels of TRAF6 polyubiquitylation.

## 6. Crosstalk between Ubiquitylation and Other UBLs (Ubiquitin-Like Proteins)

### 6.1. Neddylation of SMURF2 Decreases Its Stability

Neural precursor cell expressed developmentally down-regulated protein 8 or NEDD8 comes under the category of “ubiquitin-like protein.” Similar to ubiquitylation, NEDD8-activating enzymes E1, E2, and E3 conjugate NEDD8 onto the lysine residues of its substrates in a process referred to as neddylation. Although neddylation has been widely studied for its involvement in the activation of Cullin–RING type E3 ubiquitin ligases [31], recent studies have demonstrated neddylation of non-Cullin–RING substrates [32,33]. Recently, Shan He and colleagues have shown the effect of non-covalent binding of NEDD8 to SMURF2 and SMURF1 [34]. NEDD8 binding sites on the HECT domains of SMURF1/2 was further shown to be necessary for its ligase activity, as demonstrated by its ability for autoubiquitylation and ubiquitylation of substrates. By simulating protein interaction modes between the HECT domain and NEDD8, authors have demonstrated two regions on N-lobe and C-lobe of HECT domain as possible NEDD8 binding sites. Furthermore, mutating this region on either lobe abrogated the activity of SMURF1/2. The authors tested the consensus NEDD8 binding sites on SMURF2 and show that mutating it reduced the ability of SMURF2 to get neddylated, which in turn stabilized the protein. The study further shows the link between neddylation and SMURF2 in terms of the TGF-β pathway since mutating SMURF2 neddylation consensus sites impaired its ability to inhibit the signaling.

### 6.2. Sumoylation of SMURF2 Enhances Its Activity

Small ubiquitin-like modifier or SUMO are ubiquitin-like proteins that become covalently attached to its substrate. Similar to the process of ubiquitylation, a cascade of three enzymes, a SUMO E1 activating enzyme, SUMO E2 conjugating enzyme (e.g., Ubc9), and SUMO E3 ligases promote the covalent attachment of SUMO onto its substrates. The SENP (sentrin-specific proteases) family of desumoylases removes SUMO from protein substrates, thereby reversing the process [35,36]. Recently, SMURF2 was identified as a novel target of the SUMO pathway in NMuMG cells, a mouse mammary cell line [37]. The authors made use of NEM (*N*-ethylmaleimide) to inhibit the activity of desumoylases, which might interfere with the detection of SUMO signals. Furthermore, the detection of sumoylated SMURF2 was enhanced in the presence of NEM, indicating that SMURF2 undergoes sumoylation. Additionally, SMURF2 bound to Ubc9 and the SUMO E3 ligase, PIAS3 (among other PIAS members), which enhanced SMURF2 sumoylation most potently. The study further shows that sumoylation of SMURF2 was a reversible effect as SENP1 and SENP2 were able to remove SUMO signals from SMURF2. Using database analysis and SUMO prediction software, lysines 26 and 369 on SMURF2 were found to be major SUMO acceptor sites, as mutating either or both of the sites significantly reduced the ability of SMURF2 to get sumoylated. Furthermore, the expression of double lysine mutant (KK26–369RR) affected the ability of SMURF2 to inhibit TGF-β-induced EMT in NMuMG cells. The authors also observed that, while wildtype SMURF2 was able to reduce the levels of TβRI, the SMURF2 mutant (KK26–369RR) was impaired in its ability to do so, suggesting that sumoylation promotes its ability to induce degradation of TβRI.

### 6.3. Regulation of USP4 by Akt (Protein Kinase B) Phosphorylation

Ubiquitin-specific protease 4 (USP4) was found to regulate the TGF-β signaling through a gain of function screen [38]. cDNAs overexpressing ~27,000 genes were analyzed for their effect on TGF-β signaling with the help of CAGA–luciferase reporter assay. Through such a screen, USP4 came as one of the top hits. USP4, when overexpressed, potently enhanced TGF-β signaling. The authors further go on to show that USP4 was able to deubiquitylate the TGF-β receptor I (TβRI) directly and rescue it from proteasome-mediated degradation. However, the catalytically inactive mutant of USP4 was not able to carry out such an activity, indicating that USP4 through its catalytic site was able to function as a deubiquitylating enzyme, controlling TGF-β receptor levels at the plasma membrane. Although USP4 was shown to affect TGF-β receptors at the cell surface, endogenous USP4 staining pattern was detected mainly in the nucleus. Interestingly, the authors found out that USP4 had an Akt (protein kinase B) consensus RxRxxS(T) phosphorylation motif at Ser445. They further showed that USP4 could interact with Akt at endogenous level, and phosphorylate USP4 in vitro and in vivo. However, Akt could not phosphorylate USP4 (S445A) mutant. The authors also observed phosphorylation of endogenous USP4 upon the addition of TGF-β. While a USP4 S445A mutant was almost exclusively present in the nucleus, a phosphomimetic mutant of USP4, S445D showed some cytoplasmic localization compared to control. These results indicate that Akt phosphorylates USP4 and regulates it subcellular localization by promoting USP4 to the membrane and cytoplasm. Furthermore, the phosphorylation of USP4 by Akt was also required for it to associate with other DUBs to deubiquitylate the TGF-β receptors.

### 6.4. Regulation of Arkadia by Sumoylation

Arkadia or RNF111 is a RING domain containing E3 ubiquitin ligase, which has been previously shown to be a positive regulator of the TGF-β pathway. It has been shown to ubiquitylate and degrade SMAD7, which causes an overall increase in TGF-β signaling [39]. Additionally, Arkadia has been shown to induce degradation of SnoN and c-Ski (transcriptional co-repressors) to enhance TGF-β signaling [40]. Huaiyu Sun and colleagues have shown previously that Arkadia harbors a SUMO-binding domain (SBD) [41]. In a more recent study, they have investigated the function of SBD of Arkadia in a rather interesting context. Through immunofluorescence and mutational analysis, they show that SBD of Arkadia is critical for its localization to polycomb bodies in the nucleus [42]. They show that SBD of Arkadia is useful in targeting it to promoters, which are epigenetically silenced by polycomb bodies, and this binding through SBD could cause Arkadia to elicit both activating and repressive activities in gene regulation.

## 7. Clinical Significance and Perspective

The dichotomous nature of the TGF-β pathway has been linked to human diseases including cancer. The past few years have seen many reports on ubiquitin modifying enzymes aberrantly expressed in the TGF-β pathway. Several E3 ubiquitin ligases and DUBs have been characterized to play crucial roles in the pathway. These enzymes are being increasingly seen as potential therapeutic targets and several research groups are already screening for drugs and small molecule inhibitors to target such enzymes. Some of these enzymes have been shown to undergo other PTMs that affect its catalytic activity, substrate recognition ability, subcellular localization, etc., which ultimately influences the TGF-β pathway. The knowledge of such potential crosstalk between ubiquitylation and other PTMs would be helpful in screening for small molecule inhibitors towards these enzymes. Additionally, whether other ubiquitin enzymes undergo PTMs in the context of TGF-β pathway remain to be explored.
